# Observers Exploit Stochastic Models of Sensory Change to Help Judge the Passage of Time

**DOI:** 10.1016/j.cub.2010.12.043

**Published:** 2011-02-08

**Authors:** Misha B. Ahrens, Maneesh Sahani

**Affiliations:** 1 Gatsby Computational Neuroscience Unit, University College London, London WC1N 3AR, UK

## Abstract

Sensory stimulation can systematically bias the perceived passage of time [1–5], but why and how this happens is mysterious. In this report, we provide evidence that such biases may ultimately derive from an innate and adaptive use of stochastically evolving dynamic stimuli to help refine estimates derived from internal timekeeping mechanisms [6–15]. A simplified statistical model based on probabilistic expectations of stimulus change derived from the second-order temporal statistics of the natural environment [16, 17] makes three predictions. First, random noise-like stimuli whose statistics violate natural expectations should induce timing bias. Second, a previously unexplored obverse of this effect is that similar noise stimuli with natural statistics should reduce the variability of timing estimates. Finally, this reduction in variability should scale with the interval being timed, so as to preserve the overall Weber law of interval timing. All three predictions are borne out experimentally. Thus, in the context of our novel theoretical framework, these results suggest that observers routinely rely on sensory input to augment their sense of the passage of time, through a process of Bayesian inference based on expectations of change in the natural environment.

## Highlights

► Stimulus-timing interactions extend to stochastic “noisy” stimulation ► Stochastic stimuli reduce the variance of duration judgments ► Results support a Bayesian model in which stimuli contribute to sense of time

## Results and Discussion

### A Stochastic Change Model of Stimulus-Derived Timing

**Figure 1:**
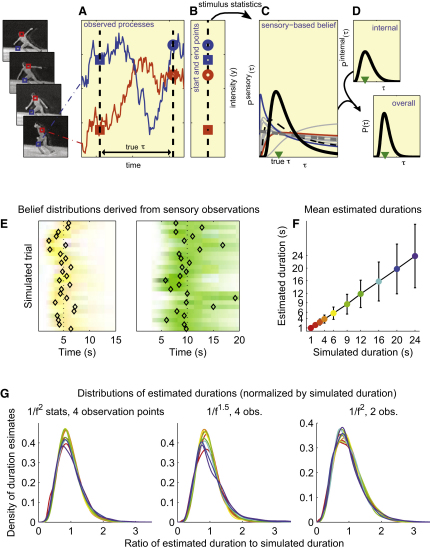
Using Snapshots of Stimuli to Judge Elapsed Time (A–D) The stochastic change model. (A) A Bayesian observer derives a sensory-based duration estimate from observation of sensory signals, such as the intensity of light falling on different points of the retina as illustrated conceptually on the left. If the observer's knowledge of natural temporal structure is limited to the second-order statistics, then its estimates will be equivalent to those of an ideal observer of stationary Gaussian processes with the same second-order statistics. The example time series {*y_i_*(*t*)} are therefore Gaussian; that is, for discrete times *t*_1_…*t_N_*, *P*(*y_i_*(*t*_1_), *y_i_*(*t*_2_),… *y_i_*(*t_N_*)) is an *N*-dimensional Gaussian distribution with mean 0 and covariance K. The *N* × *N* matrix K has elements [K]*_mn_* = exp(−λ|*t_n_* − *t_m_*|) + σ^2^δ*_mn_* with λ = 0.01 s^−1^, σ = 0.1, and δ*_mn_* = 1 if *m* = *n* and 0 otherwise. This form matched the 1/*f*^2^ power-law statistics of natural scenes [16]. Memory limitations constrain the number of observations available to the observer. Here, observations are illustrated by points at the start (squares) and end (circles) of an interval of length τ marked by the dashed lines. Later simulations used up to two further observations made at intervening times given by a Poisson process with rate 1 s^−1^ with random forgetting. (B) The discrete set of observations, or snapshots, shorn of their temporal labels, forms the basis of the sensory-derived time estimate. (C) The normalized likelihood of elapsed times *P*(observations|τ) induced by the limited observations of 12 model sensory processes (corresponding, say, to 12 well-separated points on the retina—this number was used in all simulations) and the second-order stimulus statistics. Blue and red lines correspond to the distributions induced by observations of the blue and red processes in (A); dashed black line corresponds to the distribution induced by the red and blue processes combined; gray lines correspond to the distributions induced by each of the other processes (time courses not shown); solid black line corresponds to the normalized likelihood function induced by all 12 processes together. The peak of this function gives the sensory maximum-likelihood (ML) estimate of elapsed time, which here happens to fall close to the true duration (green triangle). (D) The sensory likelihood is combined with an internal estimate *P*_internal_(τ) according to Bayes' rule to yield an integrated posterior belief *P*(τ|observations) ∝ *P*(observations|τ)*P*_internal_(τ). The internal estimate was taken to be gamma-distributed with a peak that varied across trials with a scatter consistent with the Weber law of timing [14]. Results did not depend on the details of this internal distribution. The peak of the posterior distribution yields the estimated duration in the combined model, used in the simulations in Figure 3 and Figure 4. (E–G) Modeled sensory duration estimates. (E) Example belief distributions over elapsed time, each derived from four observations of the model sensory processes with 1/*f*^2^ statistics as above and with simulated durations of 5 s or 10 s (dotted lines). Each horizontal bar represents a single belief distribution: color saturation indicates density; hue corresponds to true simulated duration as in (F). Repeated simulations show variation due to stochasticity. The peak of each distribution (black diamond) is the ML estimate for the corresponding simulated trial. (F) Mean model estimate matches true duration. Error bars indicate standard deviation of estimates. Colors of dots give key to durations in (E) and (G). (G) Density histograms of duration estimates, grouped and normalized by true durations, are scale invariant. Colors indicate true simulated duration, as shown in (F). Scale invariance is also seen for variant models with differing power spectra (middle; see Supplemental Experimental Procedures for details of the covariance function) or differing numbers of observations (right). The skewed shape of the distributions matches human results [14]. (See also Figure S1.)

Regular stimuli of known periodicity—such as the moving hands or the ticking of a clock—provide an obvious cue to the passage of time. Although the natural world is rarely so conveniently regular, stimuli with known average statistical properties can still prove informative. We constructed a simple Bayesian model in which dynamic sensation combined with knowledge of the second-order statistics of the natural environment [16, 17] to yield a sensory-based estimate of duration (Figures 1A–1C). For simplicity, we modeled a small number of abstract sensory streams using independent Gaussian processes [18] that followed the power-law statistics of natural image sequences [16]. The streams thus resembled, in their second-order statistics, luminance signals measured far enough apart to avoid correlation on a hypothetical stationary retina. A realistic model must be constrained by memory, and thus the duration estimate could not exploit the whole of the stimulus. Instead, we used a capped number of observations (or “snapshots”), with a random forgetting process eliminating older snapshots as new ones were drawn. The estimator was based on the observed change in the stimulus between these snapshots. Intuitively, little change would suggest measurements taken close together in time, whereas greater change would point to observations spaced further apart. The exact relationship is dictated by the known statistics of the stimulus. Estimates based on only second-order properties will be mathematically identical to optimal estimates based on the corresponding Gaussian processes, as in the model.

We found that individually, sensory streams were only weakly informative about the elapsed time (Figure 1C; red, blue, and gray lines), but together they constrained the elapsed time more strongly (Figure 1C, peaked black curve; Figures 1E–1G), probabilistically bracketing the true duration. Thus, even relatively few observations of naturalistic sensory processes carry sufficient information on which to base reliable timekeeping. The stochasticity of the sensory streams meant that each simulation yielded a different distribution over the elapsed time (Figure 1E; strictly, these are normalized likelihoods, because they do not yet incorporate a prior). The peak of each distribution indicates the duration most consistent with the observed snapshots of the processes. This peak was taken to represent the observer's estimate of the elapsed time (the maximum-likelihood or ML estimate). The average of these ML estimates over many repeated simulations was equal to the true simulated duration for a wide range of intervals (Figure 1F).

We also found that the distribution of ML estimates from the model scaled with the length of the duration being estimated (Figure 1G), thus matching the scalar law of biological timing [6, 14]—a property that has often proved challenging to model. The stochastic change model is robustly scalar. The three sets of histograms in Figure 1G each represent collected estimates generated using three different versions of the model with differing assumptions about the power-law scaling of the processes and the number of snapshots. The consistency of the scalar property in all cases suggests that this behavior is a general property of the framework inherited from the power-law structure of the sensory processes. The scaling holds for intervals that fall within a broad range determined by the autocorrelation structure of the stimulus but does break down eventually (see Supplemental Experimental Procedures S1.11 and Figure S1 available online).

Observers do, of course, sense the passage of time without dynamically informative stimuli, and the internal processes that underlie this ability are very likely exploited even when sensory timing information is available. Thus, the stimulus-derived estimate modeled thus far must be combined with an internally generated, stimulus-independent one (Figure 1D). If both sensory and internally derived estimates are expressed probabilistically, then the combined belief is given by the renormalized product of the sensory likelihood and internal belief. This combined distribution (now a true Bayesian posterior) will generally be more peaked—less uncertain—than either the sensory or the internal distributions alone. We chose an internal distribution that was unbiased on average, respected the scalar property, and was (by definition) independent of the stimulus type. All simulation results were qualitatively identical for different forms of the internal estimate, and whether the internal estimate was taken to be informative or not. That is, the essential features of the model predictions derived exclusively from the sensory-based estimation scheme, and not from this hypothesized internal structure.

Our model used direct observations of the sensory streams for intuitive clarity; the results of arbitrary stochastic accumulators applied to the sensory streams could be used similarly after adjusting the expected statistics for the effects of processing. Besides the limited sampling, the small set of processes and the restriction to only second-order statistics made estimation tractable [19] but meant that this scheme was necessarily an abstraction of the potential biological mechanism. Nonetheless, even this simplified model reproduced many features of human behavior, providing a realistic approximation of the true computation. The qualitative behavior of the model was surprisingly independent of the values of its parameters, obviating the need to fine tune their settings.

### Behavioral Experiments

The stochastic change model makes predictions about behavior that differ qualitatively from those of competing accounts of stimulus-induced timing effects. We tested three such predictions experimentally.

#### Experiment 1: Stochastic Bias

**Figure 2:**
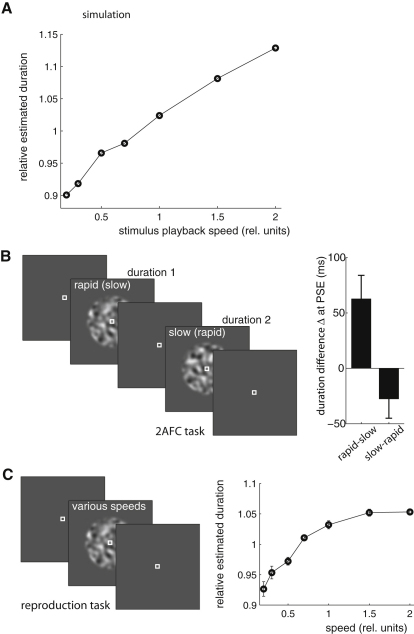
Stochastic Stimuli Can Bias Duration Judgment (A) Simulated judgments of the apparent duration of one-second-long noise stimuli played at different speeds. Mean estimated durations are shown normalized by the grand average estimate over all playback speeds. Rapid playback of the stimuli leads to overestimation. (B) Experiment 1, two-alternative forced choice (2AFC) task. Observers reported which of two smoothed Gaussian noise stimuli, presented sequentially at fixation 1 s apart, appeared to last longer. Frames of one stimulus were played rapidly and the other slowly, with the order of speeds counterbalanced. The duration of the first stimulus varied pseudorandomly between 500 and 650 ms. The second differed by an interval Δ that was adjusted by two independent staircases to find points of subjective equality (PSE) in the slow-rapid and rapid-slow conditions. Bars show the average value of Δ at PSE (n = 8; standard error shown); in both cases, the slow stimulus needed to be longer for subjective isochrony (p < 10^−6^). (C) Experiment 1, reproduction task. Subjects were asked to depress a key to match the duration of a smoothed noise stimulus presented at fixation at varying playback speeds. Durations of stimuli played rapidly were overestimated on average (n = 6; standard error shown). (See also Figure S2.)

Equal-length periodic stimuli of different frequencies appear different in duration, a finding that has been interpreted to support a counting-based contribution to timing [20]. Movies of natural movement played at unnatural speeds also bias duration judgments [21], leading to the hypothesis that observers recalibrate time to maintain physically predictable dynamics. By contrast, neither periodicity nor physical predictability plays a special role in the stochastic change model, which predicts a bias whenever the expected change induced by an ephemeral, possibly stochastic, class of stimuli differs from expectations based on the long-term spectrum. This change-based effect does indeed appear in samples from periodic processes (Figure S2) but also applies more generally (Figure 2A). Thus, the first prediction tested was that aperiodic and unpredictable stochastic stimuli should induce a systematic timing bias.

The stimuli used were samples of a rolling cloud-like spatiotemporally smooth Gaussian random process. The temporal statistics of such stimuli are captured entirely by their Fourier spectra. Using the power-law spectrum of natural scenes, we generated a sequence of video frames corresponding to regular temporal samples taken from a single random instance of the corresponding continuous noise process. The temporal statistics of the stimuli were then altered by displaying these frames at a rate either faster or slower than the sample rate, shifting the spectrum to higher or lower frequencies, respectively. A new random draw was used on each presentation, preventing observers from memorizing a particular sequence to use as a temporal reference.

Observers in the experiment saw stimuli with different temporal statistics interleaved and so were unlikely to adapt to the individual stimulus properties. Thus, the predictions of Figure 2A were based on expectations of change derived from the average spectrum of natural visual sequences. As the model stimuli changed in playback rate, biases on the order of 10% were induced in both directions. Two sets of experimental subjects were asked to report the duration of the smoothed noise stimuli in two ways. The first group reported which of two sequentially presented stimuli lasted longer (Figure 2B). The stimuli in the pair were presented at different speeds in counterbalanced order. Two interleaved staircases identified the points of subjective equality for each ordering. Compensating for presentation-order effects, slow stimuli were experienced as equal in duration to rapid stimuli about 50 ms (approximately 10%) shorter. In a second group, subjects were asked to hold down a key to reproduce the duration of the stimulus (Figure 2C). This design, used previously [20], allowed us to explore a wider range of different statistics within an experimental session at the expense of motor bias and additional variability. A range of relative biases were observed, similar to that predicted.

We conclude that the stimulus-induced “time dilation” seen in earlier studies [20, 21] is not limited to periodic or physically predictable stimulation, supporting the more general stochastic model. The empirical saturation of the effect (Figure 2C) is not seen in the simple model we implemented (Figure 2A), but, as discussed above, the sampling scheme was chosen for conceptual clarity and is deliberately unrealistic. One particular aspect neglected here is the filtering of higher temporal frequencies by the visual system [22]. In addition, decision-related behavioral effects (e.g., [1, 23]) may have shaped the measured responses. Nonetheless, the qualitative support for the model predictions, made without fine tuning the data, is strong.

#### Experiment 2: Precision

**Figure 3:**
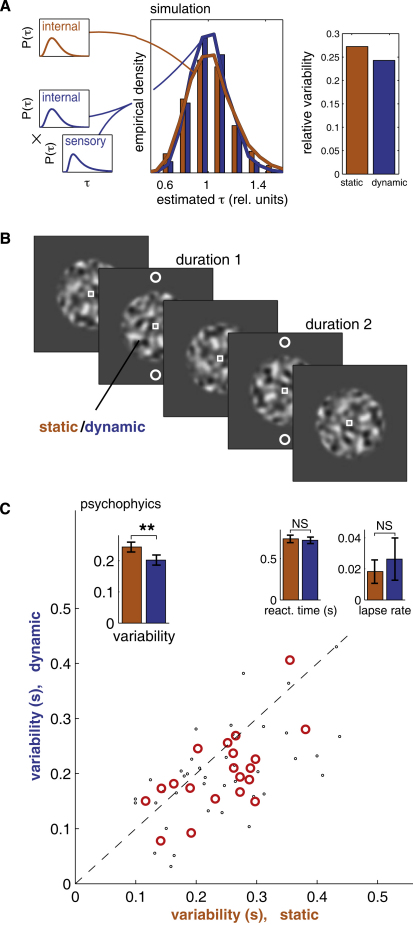
Stochastic Stimuli Reduce Variability in Duration Judgment (A) In the model, the distribution of estimates obtained by combining both internal and sensory cues (blue histogram and line; distributions obtained from 5000 simulated trials) is narrower and more peaked than that obtained from the internal estimate alone (orange histogram and line). The relative variability (right) is the standard deviation divided by the mean. (B) Design for experiment 2. A static or dynamic smoothed Gaussian noise stimulus appeared continuously at fixation. Twice in each trial, a pair of circles appeared at the edge of the noise stimulus. Duration 1 was 410 or 650 ms; duration 2 (appearing 588 ms or 50 video frames later) was a multiple of this (the range of multiples was adjusted for each subject to yield a complete psychometric curve, typically 0.45–1.8). Subjects reported whether the first or the second presentation lasted longer. Static and dynamic conditions alternated in four blocks, with the order of blocks counterbalanced across subjects. (C) Psychophysical results. Psychometric curves (n = 20) were fit by a cumulative Gaussian combined with a probability of accidentally entering an unintended response (the lapse rate, also fit to the data). The variability was defined as the reciprocal of the maximal slope of the lapse-independent psychometric curve, corresponding to the width of the Gaussian. Scatter plot and left inset: the variability during the dynamic condition was significantly lower than that during the static condition (p = 0.005, two-sided paired t test; red circles represent per-subject averages; small black circles represent each base duration for each subject; orange and blue bars in the inset are population averages for the static and dynamic conditions, respectively, with standard errors.) Middle and right insets: mean reaction times during the easiest trials were not significantly different between the static and dynamic conditions (p = 0.64), nor were the lapse rates (p = 0.73). Error bars indicate standard errors.

A key feature of the stochastic change model is that sensory information augments internal timing. If this augmentation approaches statistical efficiency, then the variability of timing estimates should fall when stimuli with known statistical temporal structure are available, even if those stimuli evolve randomly. This prediction may seem counterintuitive. It is clear that watching a clock or an hourglass can improve timing precision, but stochastic stimuli might have been expected to inject noise into the timing process; indeed, this prediction is likely to emerge from models that depend on stimulus-driven network evolution [9, 15]. In other accounts where stimulus-induced biases are incidental to changes in overall neuronal activity (e.g., [24]), variance should remain unaffected. By contrast, the integration of sensory and internal distributions in our model reduced the variability of simulated time estimates based on stochastic stimuli (Figure 3A). The exact size of this effect depended on the number of sensory streams that were tracked, but the distribution always narrowed provided the sensory estimate was not substantially biased.

We tested this prediction using a stochastic stimulus similar to that of experiment 1 (Figure 3B). This time, the smoothed Gaussian noise was present throughout a block of trials. A pair of white circles appeared above and below the noise stimulus for two nonoverlapping intervals during the trial, and subjects were asked to report which of the intervals was longer. Two conditions were distinguished by the temporal properties of the stochastic stimulus. In the static condition, a single frozen frame of the noise process remained visible and unchanged throughout the block. Because this stimulus contained no temporal cues, duration judgments had to be based entirely on internal processes. In the dynamic condition, the Gaussian noise evolved with naturalistic second-order statistics in both space and time. The noise process continued between trials without resetting; thus, details of its appearance could not be used for timing. However, its statistical properties were regular, and thus the model predicts that it would improve the precision of the estimates. Subjects viewed both static and dynamic noise stimuli extensively before the experiment began, allowing them to adapt to the statistics of the two processes.

We assessed the precision of estimates by fitting psychometric curves to the subjects' responses. Overall, estimates were less variable in the presence of the temporally random stimulus than with the stationary one (Figure 3C). To rule out changes in attention or arousal between the conditions, we analyzed reaction times (RTs) and lapse rates (estimates of inattentiveness derived from the psychometric fits) during the easiest trials. In these trials, where the second stimulus was very short, the task was equally simple under both conditions. Thus, RT changes due to differing attentional states could be distinguished from the general impact on RT of task difficulty. Neither RTs nor lapse rates on these trials were significantly different between the two conditions, suggesting that the moving stimulus had little effect on attention, motivation, or arousal (Figure 3C, insets). We conclude that visual stimuli do indeed provide usable sensory cues for time estimation, which can be combined with internal estimates to improve reliability.

#### Experiment 3: Variance Scaling

**Figure 4:**
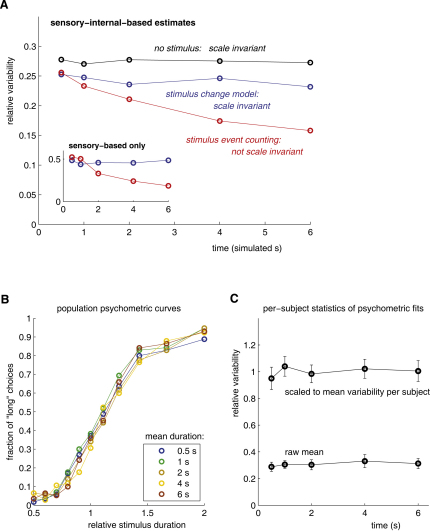
Weberian Behavior with Stochastic Stimuli Contradicts Counting Models (A) Model predictions. A model assuming an “internal” stimulus-independent estimate (black) combined with a count-based sensory estimate showed lower relative variability (standard deviation of estimates divided by true duration), violating the Weber law (red). Weberian behavior was preserved when the estimate was change based (blue). In both cases, combining the sensory-based estimate with the internal one increased precision. The distinction in variance scaling between the two models was stronger when estimates were purely sensory based (inset). The count-based model was based on the distribution *P*(τ|*N_e_*) over elapsed time τ given *N_e_* observed “events,” defined by threshold crossings. This distribution was constructed by simulating Gaussian processes over a range of possible time intervals and building an empirical histogram of event number to yield a joint table of frequencies of times and counts. The sensory *P*(τ|*N_e_*) was obtained by normalizing constant-count slices of this table and was then combined with the internal estimate as for the stochastic change model. (See also Figure S3.) (B and C) Psychophysical results. Subjects (n = 17) saw a single interval marked by the appearance of circles around a dynamic noise pattern as in experiment 2 and classified each interval as either “long” or “short.” The relative variability was independent of the mean duration, in accordance with the Weber law and with the stochastic change model. Data for (B) and (C) are the same but are shown as a population psychometric curve in (B) and as average relative variability (derived from psychometric fits to each subject) in (C). Error bars show standard errors of the means.

Despite its aperiodicity, the stochastic stimulus—and, by extension, natural sensation—might support a more elaborate event-counting scheme [1, 2, 20] based on nonlinear event detection. For instance, observers might count appearances of a region of a certain threshold size and brightness. However, the variance (not standard deviation) in the number of such events grows linearly with time, and so such a counting scheme would predict a sub-Weberian law in situations where the sensory component contributed significantly to the overall estimate. This effect was seen in simulations based on the same underlying naturalistic Gaussian processes as the stochastic change model (Figure 4A). Events were detected each time a 1/*f*^2^ Gaussian process crossed a threshold value. We assumed that observers had access to the true statistics of event occurrence and that they used this knowledge to infer a probability distribution over intervals given an event count (see Figure S3). As in the change model, this distribution combined with a probabilistic internal estimate to yield the simulated response.

The stochastic change model makes the contrasting prediction, with deviations scaling with the mean for purely sensory estimation (Figure 1G) and therefore also when sensory and internal estimates are combined (Figure 4A). We were thus able to distinguish between the models empirically. Subjects were asked to classify stimuli as shorter or longer than average within blocks of trials. Without a dynamic stimulus, subjects' choices are known to conform to the Weber law in this experiment [25]. When the same Gaussian dynamic stimulus used throughout this study was present, we found that the Weber law still held, both in the population psychometric curve and in the individual psychometric fits (Figures 4B and 4C). This ruled out an event-counting explanation for the biasing and variance-reducing effects of this dynamic stimulus. By contrast, the stochastic change model agrees with all of the experimental findings.

### Conclusion

The framework developed here provides a novel account of how stimuli interact with the perception of temporal intervals. It is based on a probabilistic formulation of the Helmholtzian [26] view of perception as unconscious inference that exploits implicit knowledge of the structure of the environment, a view that underlies many successful accounts of perceptual phenomena [27]. In our framework, temporal statistical structure in the environment provides an important cue to elapsed time. Thus, the bias induced by unnaturally structured stimuli is a counterpart to the improved accuracy gained when the environment accords with expectations. Sustained alterations of environmental statistics may lead to adaptation of an observer's model of expected change—or recalibration of their internal clocks—thus modifying the stimulus-duration contingencies. The reported locality of such adaptation [28] suggests that observers' models are sophisticated enough to capture the joint distribution over temporal change and space.

Clear experimental evidence for a dedicated physiological timing mechanism, such as a bank of specialized oscillators or integrators [6, 8], has been lacking. Consequently, many recent models of internal timing have depended on the extraction of temporal information from more general neural processes [9, 11, 15, 20, 29, 30]. Some of these models [9, 15] are based on a deterministically evolving neural network: if the network changes stereotypically and returns to baseline sufficiently slowly, then its state may provide a reliable measure of time since stimulus onset. If the evolution of this network is sensitive to the ongoing stimulus, and if the temporal estimate is closely tied to the particular network state reached, then this sort of model may be difficult to reconcile with the accuracy improvements seen in experiment 2. On the other hand, if the interpretation of network state is based on probabilistic expectations, as we have argued is the case for external stimuli, then it may be possible to reconcile our results with this type of model. Studies of the temporal evolution of neural activity have revealed an approximate underlying power law [31–33], which means that, as with natural stimuli, temporal structure in the evolution of neural systems is evident over a range of scales. Thus, a decoding scheme similar to that proposed here might also be applicable to intrinsic neural activity. As in the deterministic network view, time estimation would thus arise as a corollary to other neural computations. Most crucially, the scalar property—which has been challenging to reconcile with many past models—would arise naturally. Thus, the statistical framework proposed here may well hold the potential to further integrate the mechanisms of both internal and stimulus-derived time estimation.

## Supplemental Information

Document S1. Three Figures and Supplemental Experimental Procedures
