# Supplementary material for: Observers Exploit Stochastic Models of Sensory Change to Help Judge the Passage of Time
Source: Curr Biol. 2011 Feb 8;21(3):200–6. doi: 10.1016/j.cub.2010.12.043 (PMC3094759; doi:10.1016/j.cub.2010.12.043)
Supplement: Document S1. Three Figures and Supplemental Experimental Procedures [file mmc1.pdf]

Current Biology, Volume *21*

Supplemental Information

Observers Exploit Stochastic Models of Sensory Change to Help Judge the Passage of Time

Misha B. Ahrens and Maneesh Sahani

Supplemental Data

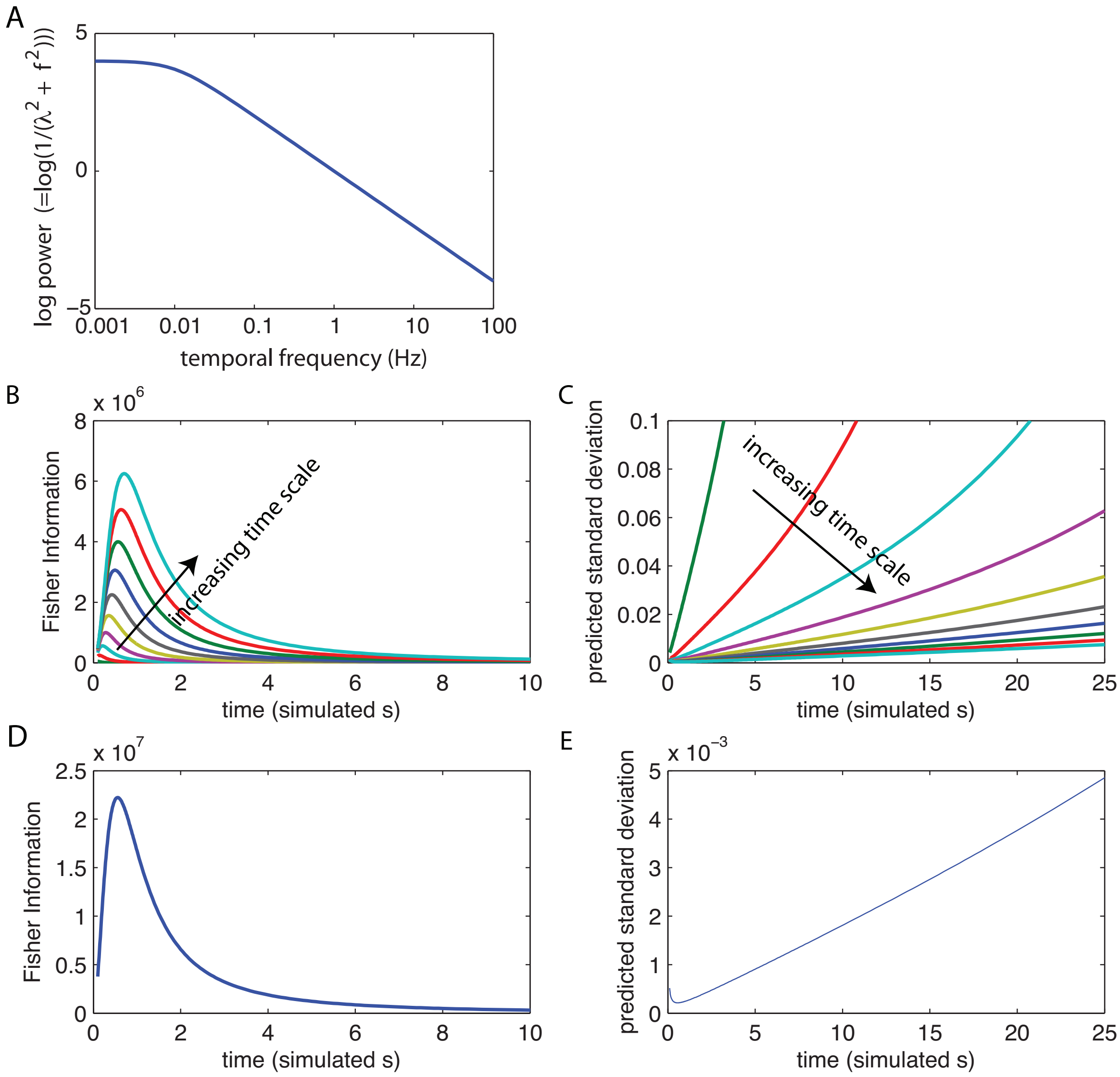

Figure S1. Related to Fig. 1. **(A) Spectrum of Ornstein-Uhlenbeck process.** Power spectrum of the model under the Ornstein-Uhlenbeck covariance function, fit approximately to the data of Dong and Atick [16]. This power spectrum determines the covariance function of the Gaussian Process used in the models. **(B-E) Origin of the Weber Law.** (B) To illustrate how the Weber law arises naturally from a multiscale Gaussian process, we used a collection of simpler Gaussian processes, each with its own time scale. For each such process (time scales 1,6,...,51 increasing in the direction of arrow), the Fisher Information (see Sec. S1.11) between two snapshots from the process and the time interval between them is shown as a function of the interval duration. (C) Predicted standard deviation of duration estimates using only one of the single-time-scale processes. The curves are initially linear (Weberian) but at larger times they increase super-linearly. Longer time scales (i.e., slower processes) produce more accurate time estimates, corresponding to the higher Fisher Information values. (D) Fisher Information between measurements of *all* the processes together and duration. This is simply the sum of all the curves in panel B. (E) The corresponding predicted standard deviation ($= 1/\sqrt{I_F}$) is approximately a linear function of time, in accordance with Weber's law.

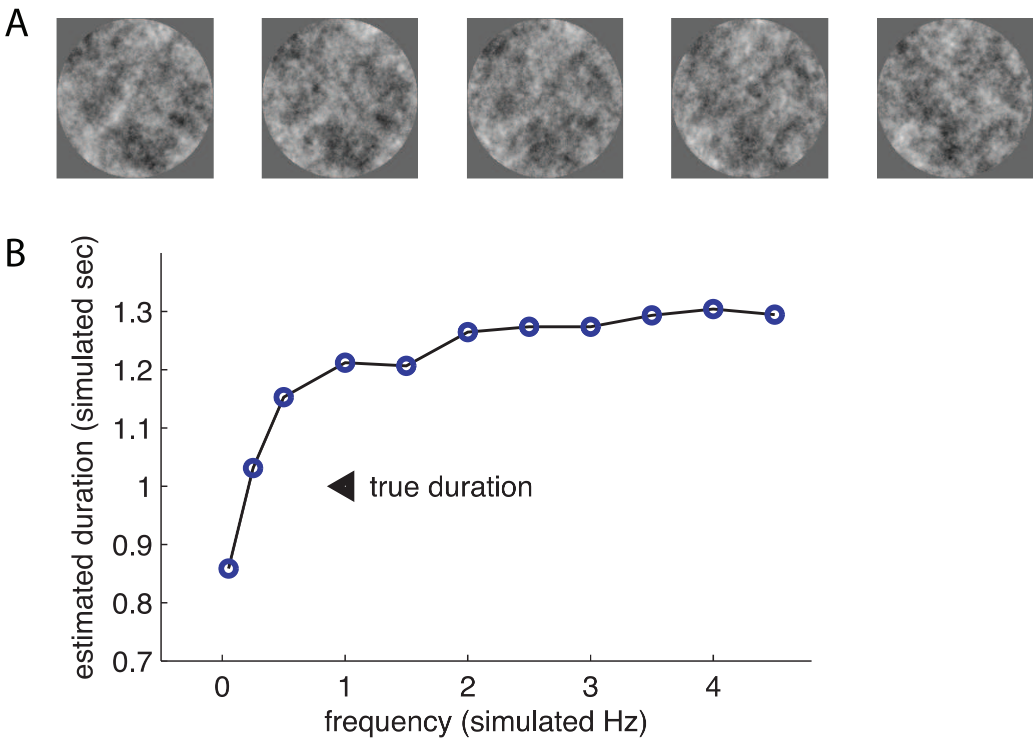

Figure S2. Related to Fig. 2. **(A) Gaussian noise stimulus.** Five example frames of the dynamic coloured Gaussian noise stimulus used for the psychophysics experiments. Interval between frames is 117 ms. **(B) Bias induced by period stimuli.** The model also reproduces time dilation for periodic stimuli. Depicted are average model time estimates for sinusoidal stimulus input. As for stochastic stimuli (Fig. 2), the model reproduces time dilation seen at higher temporal frequencies [20].

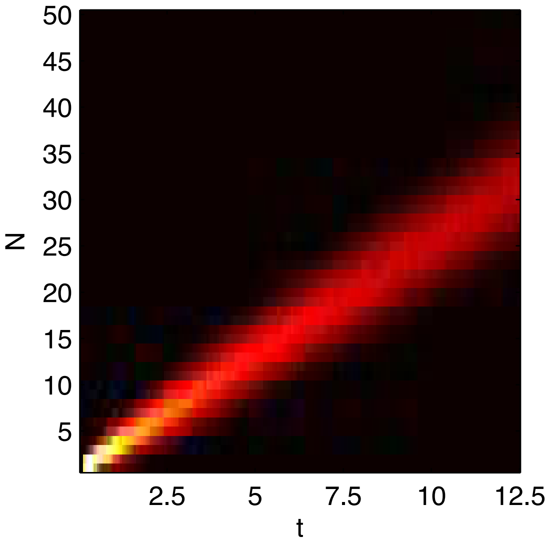

Figure S3. Related to Fig. 4. **Histogram of event counts.** The event-counting model was based on the depicted distribution between elapsed time and the number of events, which was numerically constructed. This joint distribution can be used for transforming an event-count into a distribution over elapsed time in a statistically optimal manner.

Supplemental Experimental Procedures

S1.1 Bayesian observer model

The Bayesian observer takes measurements from a number of sensory streams (or, more generally, from internally integrated processes derived from the sensory streams) and estimates the length of the time interval from which these measurements were drawn. This estimate is based on knowledge of the temporal statistics of the stimulus, expressed by the distribution $P(\text{observations}|\text{elapsed time})$ and a prior belief about the length of the interval $P(\text{elapsed time})$. This prior may be uninformative (for example, assigning equal probability to all possible intervals up to a lifetime) so that the observer's estimate is based on sensory data alone; or it may provide an independent estimate of the interval generated by internal, stimulus-independent timing mechanisms. The observer's estimate is then derived from the posterior probability distribution, which is formed by renormalising the product of these two functions, and which indicates the relative strength of the observer's belief in each possible value of the interval:

$$P(\text{elapsed time}|\text{observations}) \propto P(\text{observations}|\text{elapsed time})P(\text{elapsed time})\,. \tag{1}$$

We take the interval at which this distribution peaks to be the observer's estimate. If the prior is uninformative, then this peak will correspond to the peak of the likelihood function alone (that is, the distribution $P(\text{observations}|\text{elapsed time})$ when viewed as a function of the elapsed time for fixed values of the observations) and is therefore called the maximum-likelihood or ML estimate.

The observer model was constrained by two assumptions—first, that its knowledge of the temporal structure of the natural environment is limited to its second-order statistics; and second, that these statistics are stationary in time so that there is no statistically-preferred temporal reference point. The first of these assumptions restricts the relevant statistics of the stimulus to only the mean and covariance, the second implies that the mean is constant. Thus, the stimulus-driven duration estimates must be based on the covariance structure of the sensory processes alone. As a result, estimates derived under these assumptions are equivalent to those obtained by an *ideal* observer working with measurements of a set of stationary Gaussian processes (GPs) [18] (see also Sec. S1.2) whose second-order statistics matched those of natural scenes. Thus in the mathematical development below we assume that the observed sensory processes are indeed Gaussian and stationary, and carry out an ideal observer analysis under these conditions.

The model used 12 independent sensory processes. Although realistic sensory input, such as the pattern of light falling on the retina, is correlated in space as well as in time [16], it is always possible to spatially decorrelate the processes using an instantaneous linear transform (sometimes called the *whitening* or *sphering* transform). Loosely, the independent processes might be identified with the time-series of the luminance at several points of visual space separated sufficiently to be uncorrelated. More exactly, but perhaps less intuitively, they would be given by weighted sums of the different pixel time-series, with the weights arranged so that the resultant sums were uncorrelated. Each individual independent process, $y_i(t)$, $i = 1 \ldots 12$, had zero mean and a covariance function $K_i(\tau)$ which, in most simulations, was of Ornstein-Uhlenbeck (OU) form:

$$K_i(\tau) = e^{-\lambda|\tau|} + \sigma^2\delta(\tau)\,, \tag{2}$$

where, $\delta(0) = 1$ and $\delta(x) = 0$ for $x \neq 0$. The constants were set to $\lambda = 0.01$ s and $\sigma = 0.1$ (arbitrary units; the significant factor being the ratio of the two covariance components). This covariance function generates a GP with $1/f^2$ power spectrum over a wide range of time scales. These choices were motivated by measurements of natural image statistics [16]; other choices did not change the qualitative behaviour of the model. The one exception to the OU covariance was in the simulations used to generate the middle panel of Fig. 1G; these are described below in Sec. S1.3. Together, the assumed mean and covariance function fully specify the joint Gaussian distribution over observations of each processes at N specified times:

$$P(y_i(t_1), y_i(t_2), \ldots y_i(t_N)|t_1 \ldots t_N) = \mathcal{N}(0, \mathsf{K}_i)\,, \tag{3}$$

where the $N \times N$ matrix K_i has elements $[\mathsf{K}_i]_{mn} = K_i(t_n - t_m)$, and $\mathcal{N}(0, \mathsf{K}_i)$ denotes an N-dimensional Gaussian distribution of mean 0 and covariance K_i.

We assumed that observations were made at the beginning and end of the interval to be timed and at up to two intervening times. The intervening sample times were chosen according to a homogeneous Poisson process with rate 1 s^{-1} with random forgetting. That is, potential sample times were drawn in order according to the Poisson process and corresponding sensory measurements stored. If two measurements were already in memory, then one of these (chosen with equal probability) would be "forgotten" to make room for the new one. The net effect is that samples were drawn at times given by a reversed exponential distribution with decay rate $1/2$ s^{-1}. The likelihood function $L_{\mathsf{sensory}}(\tau) = P(\text{observations}|\text{elapsed time})$ for elapsed time τ was then

$$L_{\mathsf{sensory}}(\tau) = \int_0^\tau \int_0^\tau dt_1\, dt_2 \prod_{i=1}^{12} P\big(y_i(0), y_i(t_1), y_i(t_2), y_i(\tau)|t_1, t_2, \tau\big)\; P_{\mathsf{exp}}(t_1) P_{\mathsf{exp}}(t_2)\,, \tag{4}$$

where $P_{\mathsf{exp}}(t) = \frac{1}{2}\exp\big(-\frac{1}{2}(\tau - t)\big)$. The product represents the combined probability of observations from the 12 independent processes; the integrals reflect the fact that the intervening sample times are unknown and so all possible values for those times must be considered. The initial time has been set to 0 without loss of generality as the processes are assumed to be statistically stationary. This likelihood function was normalisable and so, suitably normalised, was treated as the sensory belief without incorporation of an explicit uninformative prior.

The simulations of Figs. 3 and 4 required the incorporation of an internal estimate of elapsed time $P_{\mathsf{internal}}(\tau)$. The internally generated distribution is taken to have the shape of a Gamma distribution whose peak varies across trials with a scatter consistent with the Weber law of timing [14], described in further detail in Sec. S1.5. (The Weber property of the internal model was dictated by known results; as seen in Figs. 1G and 4A, the Weberian nature of the sensory estimate derives from the sensory likelihood term and does not depend on this choice for the internal estimate.) The simulation of Fig. 2A incorporated the same internal estimate, although the same qualitative result is obtained from the sensory estimate alone, in which case the relative estimated duration would scale linearly with the playback speed. We also experimented with a different choice of internal-estimate distribution, based on a Gaussian likelihood with scaled standard deviation, and all results remained qualitatively identical (data not shown).

S1.2 Stationary Gaussian processes

The stochastic change timing model was based on second-order sensory statistics, and could thus be formulated as acting optimally on stationary and Gaussian sensory processes. We briefly review the definition and use of stationary GPs. Further details about GPs may be found in Supplementary refs [18, 34].

A one-dimensional stationary GP is a probability measure over functions $y : \mathbb{R} \to \mathbb{R}$, which is fully parametrised by a constant mean (here taken equal to 0 without loss of generality) and a covariance function $K : \mathbb{R} \to \mathbb{R}$ giving the covariance between the function values at two points with a given separation. We take the domain of the functions y to be time t. If a function drawn from the GP is sampled at a set of discrete times $[t_1, t_2, \ldots, t_N]$, then the sampled function values $[y(t_1), y(t_2), \ldots, y(t_N)]$ jointly follow a multivariate Gaussian probability law:

$$p\Big([y(t_1), \ldots, y(t_N)] \;\Big|\; [t_1, \ldots, t_N]\Big) = \det[2\pi \mathsf{K}]^{-1/2} \exp\left\{ -\frac{1}{2}[y(t_1), \ldots, y(t_N)]\mathsf{K}^{-1}[y(t_1), \ldots, y(t_N)]^{\mathsf{T}} \right\}\,, \tag{5}$$

where the covariance matrix depends on the GP covariance function and the sample time points, $\mathsf{K}_{ij} = K(t_i - t_j)$. The covariance function determines the expected spectrum of the GP-generated functions, and thus their smoothness. For instance, if t_2 were close to t_3, then K_{23} might be relatively high, in turn causing $y(t_2)$ and $y(t_3)$ to tend to have similar values.

In practice, sampled observations of draws from a GP can be generated with arbitrary temporal precision by discretising the time-axis, and generating the corresponding function values from a Gaussian distribution as above (as in Fig. 1A of the main text). Equivalently and more compactly, one can sample the functions

only at the observation times; equation 5 still applies. This method was used in all simulations, using observations times $(0, \tau)$ in the two-observation case, and $(0, t_1, t_2, \tau)$ in the multiple-sample case. The first time point could be set to zero without losing generality because the processes are (statistically) time-translation invariant.

S1.3 General covariance functions

For simulations in which the power spectrum did not follow a $1/f^2$ law (which were used to check the robustness of the model to perturbations in its parameters), we defined the covariance function using a weighted sum of squared exponentials, each characterised by its own time scale l_i, again combined with white noise,

$$K(\tau) = \frac{1}{Q} \sum_i l_i^p \exp\left(-\frac{\tau^2}{2l_i^2}\right) \; + \; \sigma^2 \delta(\tau)\,. \tag{6}$$

The time scales l_i ranged from 0.1 to 50 simulated seconds in steps of 0.1. The expression is normalised by $Q = \sum_j l_j^p$. The properties of the squared-exponential covariance function (that is, each individual term of the sum) are well-characterised. The scalar p determines the relative weighting of each time scale, and thus the power spectrum of the processes: suitable choices yielded processes whose statistics followed power-laws: For instance, setting $p = 0$ resulted in an approximate $1/f^2$ power spectrum, while $p = 1$ gave a $1/f^3$ spectrum, and $p = -0.5$ a $1/f^{1.5}$ spectrum. By varying p we could thus test how the model behaved with different statistical structure.

The term $\sigma^2\delta(\tau)$, where $\delta(0) = 1$, and $\delta(\tau) = 0$ for $\tau \neq 0$, represents instantaneous noise or observation noise. The noise scale σ was set to 0.1 in the simulations shown; other choices of this scale led to qualitatively identical results.

S1.4 The OU covariance and natural scene statistics

We know that the temporal power spectrum of natural scenes is broadly proportional to $1/f^2$ for temporal frequency f. The power spectrum and covariance function of a stochastic process are Fourier transforms of one another. Thus, the OU covariance function $\exp(-\lambda|\tau|)$ generates a power spectrum that is proportional to $\frac{1}{\lambda^2+f^2}$. This is indeed close to $1/f^2$ for values of f that are substantially greater than λ. At lower frequencies, the OU power spectrum saturates to the value $1/\lambda^2$; but the same must, of course, be true of the natural statistics (otherwise the mean brightness of images could grow unboundedly large). Thus by choosing λ appropriately, we can approximate the power spectrum of natural scenes.

What is this value of λ? Dong and Atick [16] report that the temporal power spectrum of natural scenes follows a power law (approximately $1/f^2$, or a slightly lower power) which holds *at least* down to a temporal frequency of 0.1 Hz, so that we conclude that λ is smaller than 0.1. Here, we approximated $\lambda = 0.01$ (which ensures that the Weber property holds for intervals up to about 100 s, see Sec. S1.11). The resulting model temporal power spectrum is shown in Fig. S1A.

The additional term ($\sigma^2\delta(t)$) in equation 2 reflects a small component of temporally-independent variability introduced into the GP to model instantaneous noise in the observation process for the sensory stream.

S1.5 The internal time estimate

For the simulations shown in Figs. 3 and 4, we included an internally generated belief over elapsed times, as illustrated in Fig. 1D. We assumed that this belief—a function of the elapsed time τ—had the shape of a Gamma distribution:

$$P_{\text{internal}}(\tau) \propto \frac{\tau^{k-1}}{\Gamma(k-1)} \exp(-\tau/\theta)\theta^{-k}\,. \tag{7}$$

with the parameter k held fixed to 40 and θ varying from trial to trial. Thus, θ served as a sufficient statistic for the internal process(es) upon which the internal estimate was based. The expression above, which is, in fact, a properly normalised distribution on θ, corresponds to θ being drawn from an inverse-gamma distribution

with mean $\tau/(k-2)$ and standard deviation $\tau/(k-2)\sqrt{k-3}$. As the peak of the likelihood is, in turn, proportional to θ, this means that the maximum likelihood internal estimate under these assumptions obeys both the proportionality and Weber laws of timing. Our results were qualitatively independent of the details of this internally generated belief over elapsed time; we also tried a normally distributed internal belief, with equivalent results. Note that Figs. 1E-G, demonstrating the scalar property of the stochastic change model, were generated using only the sensory-based ML estimate.

S1.6 Implementation of the number-of-events model

We reasoned that, if subjects were to count stimulus events while watching the smoothed Gaussian noise patterns, then the most likely stimulus feature corresponding to an "event" would be the appearance of a transient bright spot on the display. We therefore generated model Gaussian processes and took an event to occur when the process value crossed above a threshold.

In order to turn event counts into a sensory-based distribution over elapsed time, we numerically constructed the joint distribution $P(\text{number of events, elapsed time})$ or $P(N, t)$ by finely discretising the t axis, and, for every value of t, generating many processes and counting the number of events N. This joint histogram $H(t, N)$ is depicted in Fig. S3.

Next, to simulate a given trial, we generated a Gaussian process of duration t_{true}, and counted the number of threshold crossing events N_{trial}. The corresponding slice of the histogram $H(t, N_{\text{trial}})$, appropriately normalised, provided a likelihood function over the elapsed time t based on this count. The value of t for which $H(t, N_{\text{trial}})$ was greatest corresponded to the count-based ML estimate of duration. The likelihood function could also be combined with the internal distribution over elapsed time, exactly as in the "change" model, with the peak of the resulting posterior distribution giving the combined estimate. The distribution of estimates was constructed by repeating this process many times, and the relative variability computed, as shown in Fig. 4A.

The single parameter in this model is the threshold, the crossing of which defines an "event". Setting the threshold low implies a high event-rate, with relatively fine-grained information available for timing. Conversely, a high threshold implies a low event-rate, and little information for timing. We set the threshold so that the sensory-induced increase in accuracy of the model was approximately matched to that of our subjects (cf. Fig. 3C and Fig. 4A) at a duration of around 1 simulated second. This lead to an event-rate of roughly 2.5 per second.

S1.7 Smoothed Gaussian noise stimuli

Spatio-temporally-smoothed Gaussian noise, also called "coloured" Gaussian noise, was generated using MATLAB (Mathworks, Natick, MA). Conceptually, smoothed or coloured Gaussian noise can be produced by convolving independent Gaussian noise with an appropriate filter. Equivalently, a sample can be generated in the Fourier domain, by combining a suitable amplitude spectrum with uniformly random phase angles and transforming to space-time through an inverse 3-dimensional Fourier transform. The amplitude spectrum was

$$a(f_x, f_y, f_t) = 1/(1 + \alpha f_x^2 + \beta f_y^2 + \gamma f_t^{1.7}) \,, \tag{8}$$

where f_x, f_y and f_t are the two spatial and one temporal frequency respectively. This choice led to a power spectrum that was approximately $1/f^2$ in each of space and time separately, in approximate accordance with natural scene statistics. We used $\alpha = \beta = 100$ and $\gamma = 400$. Fig. S2A shows five sequentially ordered frames from the stimulus.

The frequencies $f_{x,y,t}$ are determined by the x and y ranges (in pixels) and the temporal frequency of the monitor, therefore they take on the values $f_x = [0, 1, 2, \ldots, x_{\text{max}}/2, \ldots, 2, 1]$ (with similar ranges for f_y and f_t), where $[x_{\text{max}}, y_{\text{max}}, t_{\text{max}}] = [220, 220, 1200]$, measured in x-pixels, y-pixels and video-frames respectively.

In Experiment 1, we used two additional stimuli in the 2AFC task, which were obtained from the original by bandpass filtering to either a low spatial frequency range (near 0.6 cycles/degree) or a mid-frequency range (3 cycles/degree). The results were significant ($p < 0.01$) in each separate case, thus the reported effect also holds for stimuli with limited spatial frequency content. We also tested a stimulus in which the amplitude

spectrum had a slightly different form so the spectrum was separable in (x, y) and t but the temporal spectrum was approximately equal, again with no significant change in results.

S1.8 Experiment 1

2AFC task. Stimuli were spatio-temporally smoothed Gaussian noise with a band-limited spatio-temporal spectrum, displayed in a circular area 5 degrees of visual arc in diameter, on a CRT monitor with an 85 Hz refresh rate and linearised greyscale. Details of the noise stimuli are described in Sec. S1.7 above. On each trial, two stimulus segments were presented successively (Fig. 2B), and observers were asked whether the second segment was longer or shorter in duration than the first. The duration of the first segment, t_1, was pseudo-randomly chosen between 500 and 650 ms; the second began 1 s after the offset of the first, and had a duration of $t_2 = t_1 + \Delta$. (Although the just noticeable difference is not constant for all t, the range of t_1 was sufficiently small that the use of an additive offset had little adverse impact.) The ratio between the speeds of the first and second segments was either 1:1.7 in the slow-rapid condition, or 1.7:1 in the rapid-slow condition. Conditions were pseudo-randomly interleaved, and counterbalanced so as to remove the known effect of presentation order on duration judgements [35]. After each trial, Δ was adjusted using a staircase procedure to find the point of subjective equality for the slow-rapid and the rapid-slow conditions. As the task quickly became difficult, one in 10 trials (randomly chosen) was made easier by setting Δ = 250 ms. These trials were discarded from the analysis. Trials 20-41 were analysed by averaging, per subject and separately for the slow-rapid and rapid-slow conditions, the points at which the staircase changed direction [15, 36]. A two-sided t-test on the averaged data verified that the offset Δ at which the two stimuli were judged to be of equal length in the rapid-slow condition was larger than in the slow-rapid condition. No feedback was given to the subjects.

Reproduction task. We followed the design described in [20]. The same stimulus as in the 2AFC experiment was presented once per trial, for between 0.6 and 2 seconds, at a variety of playback speeds (Fig. 2C). Once the stimulus had extinguished, subjects were cued to hold down a button for the same duration as the stimulus. The subjects did not receive feedback.

S1.9 Experiment 2

A static or dynamic noise patch (subtending 4 degrees of visual angle on a 85 Hz CRT) was continuously visible throughout each block. Two stimuli appeared, consisting of pairs of small circles displayed above and below the noise patch, and subjects were asked which of the two appearances was longer, in a 2AFC task. (The circles were faded in, and at the end of the interval faded out, linearly over a period of 70 ms.) The first duration was one of the two base durations 410ms and 650ms (pseudo-randomly interleaved), or 520ms and 820ms (for six randomly chosen subjects). The second duration was a multiple, x, of the base duration. The range of x was adjusted per subject, and typically covered 0.45-1.8.

After a training period and an initial psychometric fit for calibration of the x-range, each subject performed 400 trials over the course of four blocks. The blocks alternated between the static and dynamic conditions, with the condition of the first block randomised. Again, no feedback was given. Psychometric curves were fit to the two base durations. These curves were fit separately for each block of trials, in order to prevent non-stationary behaviour across the sessions from affecting the estimates of slope. Subjects tired rapidly due to the difficulty of this task, and so we performed relatively short experiments with relatively many subjects.

The psychometric curves were parametrised as

$$l(x) \sim \text{Binomial}\left[n(x), \epsilon/2 + (1-\epsilon) \cdot (1 + \text{erf}(\sqrt{\pi} \cdot s \cdot (x-m)))/2\right], \tag{9}$$

where $x = \frac{\text{duration 2}}{\text{duration 1}}$, $l(x)$ is the number of "second duration is longer" responses, $n(x)$ is the number of trials at point x, s is the slope, m is the midpoint, ϵ is the lapse rate, and $\text{erf}(\xi) = \frac{2}{\sqrt{\pi}} \int_0^{\xi} e^{-t^2}\ dt$ is the error function. After fitting m, s and ϵ by maximising the likelihood under the Binomial model, the variability was defined to be $\frac{1}{\sqrt{2\pi}s}$ (the standard deviation of the Gaussian distribution corresponding to the erf component of the psychometric curve). These variabilities were averaged between the two base durations for each subject,

yielding one variability value for the static condition, and one for the dynamic condition for each subject. The means of these distributions were compared using a two-sided t-test. ($N = 20$.)

Controls As explained in the main text, we compared reaction times and lapse rates to obtain a measure of attention or motivation. The average reaction times during the easiest trial conditions were not significantly different between the static and dynamic conditions ($p = 0.64$; Fig. 3C, insets). These easiest conditions, as assessed by average fraction of correct responses, were those in which the duration of the second stimulus was shortest. Responses to these trials were almost all correct and we reasoned that any difference in reaction times would signal differences in attention, arousal or motivation between the dynamic and static conditions. For more difficult conditions, reaction-time differences would be more directly related to task difficulty, and therefore not suitable as an independent measure of attention. We also compared the lapse rates, derived from the psychometric fits. (Fig. 3C, right inset). There was no significant difference between the conditions ($p = 0.73$), again suggesting that there was no difference in attention.

S1.10 Experiment 3

To test whether or not timing behaviour remained scale-invariant (*i.e.*, consistent with the Weber law of timing) in the presence of the smoothed Gaussian noise stimulus, we adapted an experimental design that had previously been used to show scale-invariance in human timing judgements made in the absence of dynamic stimuli [25].

The experimental session was divided into blocks. In each block stimuli were presented with a fixed "average duration". On each trial within the block, the stimulus duration was a multiple (see x-axis of Fig. 4B) of this average duration. Subjects were instructed to classify each stimulus as either "short" or "long" when compared to the range of durations seen on previous trials within the current block. They were not given feedback about their answers. At the start of each block, the "average duration" stimulus was shown once, to provide a reference for the first few trials. The data were analysed by plotting the population psychometric curve (where all answers are collapsed across subjects, Fig. 4B), and by fitting psychometric curves to each individual subject's data, as described in the analysis of Experiment 2, and then analysing the average variability (Fig. 4C, variability $\propto$ 1/slope). Both analyses showed that the Weber law still held in the presence of a dynamic stimulus.

S1.11 The origin of the Weber law

We derive an analytical expression for the standard deviation of interval estimates, as a function of the true interval, based on the Fisher Information. The Cramér-Rao bound states that the standard deviation of time estimates is at least as large as $1/\sqrt{I_F(\tau)}$, where the Fisher information I_F is

$$I_F(\tau) = -\left\langle \frac{\partial^2 \log P(\{\mathbf{y}\}|\tau)}{\partial \tau^2} \right\rangle_{\mathbf{y}} . \tag{10}$$

In the two-sample case (which we analyse in some detail here; the multiple-sample case is not analytically tractable but, as suggested by Fig. 1G, may be qualitatively equivalent), this equals

$$\frac{N}{2}\mathsf{Tr}\left[\mathsf{K}^{-1}\frac{\partial \mathsf{K}}{\partial \tau}\mathsf{K}^{-1}\frac{\partial \mathsf{K}}{\partial \tau}\right] , \tag{11}$$

where K is the covariance matrix. We found that the Cramér-Rao bound is tight, so that $\sigma_{\text{time estimates}} = 1/\sqrt{I_F(\tau)}$.

This analytical expression of the standard deviation as a function of time allows us to assess the reasons for the Weberian behaviour of model estimates. First, when the Gaussian processes follow Ornstein-Uhlenbeck statistics, so that the covariance function is $K(\tau) = \exp(-\lambda|\tau|)$, this expression (working through the matrix

multiplications and taking the trace) equals

$$\sigma = \frac{(1+\sigma^2)^2}{\delta}\exp(2\lambda\tau) - \frac{1}{\lambda} \tag{12}$$

$$\approx \frac{1}{\lambda}(\exp(2\lambda\tau) - 1) \tag{13}$$

$$\approx 2\tau + \mathcal{O}(\lambda^2\tau^2)\,. \tag{14}$$

This means that the Weber law is approximately satisfied until $\lambda^2\tau^2$ becomes large. The value of λ inferred from measured natural scene statistics [16] is 0.1 or smaller (see "Fitting the model to natural scene statistics"). This means that the "change" model obeys the Weber law until *at least* 10 seconds. In our simulations we chose $\lambda = 0.01$.

A complementary way to gain intuition for why the Weber law holds for the change model is to consider the different time scales that are present in $1/f^2$ processes. Suppose these time scales are artificially separated into different processes — that is, instead of having a collection (e.g. image pixels) of processes following $1/f^2$ statistics and each containing many time scales, we have a collection of processes that each contain a different but narrow range of time scales, so that some are slow (smooth) and some are fast (relatively jagged). This can be done by assigning a different covariance function to each process, which we took to be

$$K(\tau) = \exp(-\frac{\tau^2}{2l^2}) \tag{15}$$

(compare this to the terms that comprise equation 6). When l is small, the process will contain small time scales, and look jagged; when l is large, the process will contain long time scales and look smooth.

Now, the Fisher information for each of these processes provides intuition for the origin of the Weber law. Fig. S1B-E shows the Fisher information as a function of τ for each time scale l separately ($l = 1, 6, 11, \ldots, 51$). From this, it is evident that long time scale processes contain information about long intervals (i.e., large values of τ) as well as short intervals (small values of τ), whereas short time scale processes only contain information about short intervals. Thus, information about small intervals accumulates compared to information about long intervals, leading to higher precision at small intervals. Equivalently, the longer the interval, the more information is lost because fast processes are not informative anymore. The precise way in which this happens makes the Weber law quite independent of the details of the statistics of the process (see Fig. 1G). This analysis suggests that the temporal Weber law, or scale invariance, is a very general property of this type of system.

S1.12 Simulation of dilation with periodic stimuli

Kanai et al.[20] performed an experiment similar to our Experiment 1, in which observers judged the duration of moving sinusoidal gratings of different temporal frequencies. Higher temporal frequencies resulted in larger estimated durations, matching the results that we found with stochastic stimuli. The stochastic change model reproduced the results from the earlier study, as is shown in Fig. S2B.

Supplemental References

34. C. E. Rasmussen and C. K. I. Williams. *Gaussian Processes for Machine Learning*. MIT Press, Cambridge, MA, 2006.
35. L. G. Allan. The time-order error in judgments of duration. *Canadian Journal of Psychology*, 31:24–31, 1977.
36. H. Levitt. Transformed up-down methods in psychoacoustics. *J. Acoust. Soc. Am.*, 49(2):467–477, 1971.
37. P. Dayan and L. F. Abbott. *Theoretical Neuroscience*. MIT Press, Cambridge, MA, 2001.
